# Tailings microbial community profile and prediction of its functionality in basins of tungsten mine

**DOI:** 10.1038/s41598-019-55706-6

**Published:** 2019-12-20

**Authors:** Ana Paula Chung, Carina Coimbra, Pedro Farias, Romeu Francisco, Rita Branco, Francisco V. Simão, Elsa Gomes, Alcides Pereira, Maria C. Vila, António Fiúza, Martin S. Mortensen, Søren J. Sørensen, Paula V. Morais

**Affiliations:** 1 Centre for Mechanical Engineering, Materials and Processes and Department of Life Sciences, University of Coimbra, 3000-456 Coimbra, Portugal; 2 Centre for Earth and Space Research and Department of Earth Sciences, University of Coimbra Pólo II, 3030-790 Coimbra, Portugal; 3 Department of Mining Engineering and Research Centre on Environment and Natural Resources, Faculty of Engineering, University of Porto, 4200-465 Porto, Portugal; 4 Section of Microbiology, Department of Biology, University of Copenhagen, 2100 Copenhagen, Denmark; 5 Present Address: Research Centre for Economics and Corporate Sustainability (CEDON), Department of ECON-CEDON, Faculty of Economics and Business (FEB), Katholieke Universiteit Leuven (KU Leuven), Brussels Campus, Leuven, Belgium

**Keywords:** Environmental microbiology, Microbial communities

## Abstract

In a circular economy concept, where more than 300 million tons of mining and quarrying wastes are produced annually, those are valuable resources, supplying metals that are extracted today by other processes, if innovative methods and processes for efficient extraction of these elements are applied. This work aims to assess microbiological and chemical spatial distribution within two tailing basins from a tungsten mine, using a MiSeq approach targeting the 16S rRNA gene, to relate microbial composition and function with chemical variability, thus, providing information to enhance the efficiency of the exploitation of these secondary sources. The tailings sediments core microbiome comprised members of family *Anaerolineacea* and genera *Acinetobacter*, *Bacillus*, *Cellulomonas*, *Pseudomonas*, *Streptococcus* and *Rothia*, despite marked differences in tailings physicochemical properties. The higher contents of Al and K shaped the community of Basin 1, while As-S-Fe contents were correlated with the microbiome composition of Basin 2. The predicted metabolic functions of the microbiome were rich in genes related to metabolism pathways and environmental information processing pathways. An in-depth understanding of the tailings microbiome and its metabolic capabilities can provide a direction for the management of tailings disposal sites and maximize their potential as secondary resources.

## Introduction

In a circular economy concept where over 300 million tons of quarrying and mining wastes are produced annually^1^, those are a valuable resource, supplying not only the demand for metals but also promoting recycling, minimizing harmful waste, dissipation and hazards. Innovative methods and processes for the efficient extraction of these elements from secondary sources are the focus of many projects nowadays (https://ec.europa.eu/research/environment/pdf/h2020_projects_circular_economy_2016-2018.pdf). Traditionally, raw materials are obtained through the extraction and processing of high-grade ore deposits by conventional mining methods. The mining techniques efficiency for metal recovery has varied in time, mostly driven by economic sustainability considerations. Consequently, large quantities of metals have been discarded to tailings basins, often containing concentrations above the minimum grade required for exploitation by mining companies. Tungsten is considered by the EU a critical raw material (CRM), as it raises strategic concerns relative to the security of the supply chain necessary for the EU economy^2^. Panasqueira mine, Portugal, is one of the largest operating tungsten mines in the Market Economy Countries. Until now, the Portuguese mine of Panasqueira has produced several millions of tons of residues during its almost 120 years of operation. Some of the residues consist of a finely ground material produced by the ore processing plant. These materials may have interesting grades in tungsten and other metals depending on the efficiency of the technologies applied throughout the life span of the mine. The mine residues have been deposited in two tailing basins. The first basin was closed in 1985 and only the new one is currently receiving the mine tailings.

Despite the environmental toxicity, several reports describe the diversity of the microbial communities in mine tailings which play important ecological roles^3,4^. To thrive in such harsh conditions, microorganisms acquired several strategies to reduce metal toxicity. Some of these involve transport and bioimmobilization of metals by modifying their solubility and oxidation state, therefore, increasing or decreasing their availability in the environment^5^. Changes in microbial populations may be used as an indicator for environmental modifications that affect the fate of metals. Moreover, characterization of microorganisms with the potential to be applied in biosolubilization, biomineralization and bioaccumulation are needed to develop novel strategies for the recovery of metals from mine tailings as part of a circular economy.

Effective *in situ* utilization of the mine tailings, which are low-grade materials, as secondary sources of metals will benefit from the uncovering of the composition and functional potential of the bacterial communities throughout a depth vertical profile. With the developments in high-throughput metagenomics sequencing, it is now possible to characterize the uncultured microbial taxa and to ascertain their ecological role^6^. Exposure to metals can negatively affect microbial diversity and biomass^7,8^ and inhibit microbial functions^9^. Mine tailings are characteristically oligotrophic environments and loaded with toxic metal(loid)s, making them challenging for microorganism survival^10,11^. Literature indicates that microorganisms can impact the cycling of Fe and S, since they affect the release of metals and sulfur to the environment^12,13^.

This work assessed the microbiological and chemical spatial distribution of two waste deposits with different ages of a tungsten mine, using a targeted 16S rRNA gene sequencing, for the determination of the microbial composition and relate them to the chemical variability. The metabolic functions of the microbiomes in this harsh environment were also predicted using a computational approach (PICRUSt software) to provide useful inside of these uncharacterized communities.

The outcomes of this study will provide information to enhance the efficiency of the exploitation of these secondary sources. The overall goals of this study are to understand: (*i*) the bacterial diversity of the community in the basins, (*ii*) the metabolic potentials of the indigenous microbiomes predicted by computational approach and (*iii*) the correlation of the metabolic potential with the mine tailings chemical composition.

An in-depth understanding of the tailings microbiome and its metabolic capabilities can provide a direction for the management of tailings disposal sites and maximize the potential of tailings as secondary raw materials sources.

## Materials and Methods

### Study area

**Figure 1:**
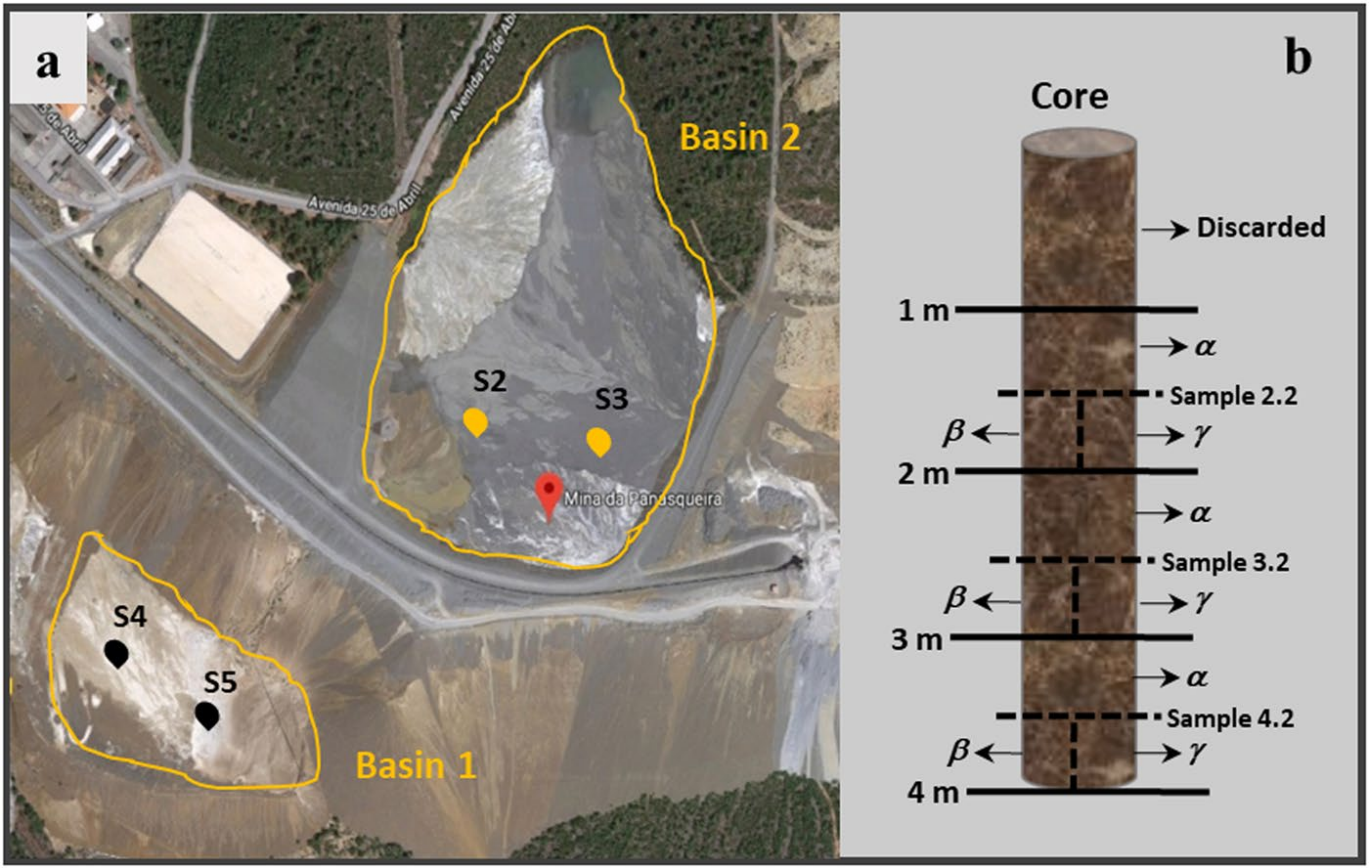
Geographical location of the sampling sites and schematic representation of the collected cores in Panasqueira mine, Portugal. (**a**) The sampling was performed in Basin 1 and Basin 2; four boreholes were sampled, S4 and S5 in Basin 1 marked with black dots and S2 and S3 in Basin 2, marked with yellow dots, respectively. (**b**) Cores with 4 meters long were collected from the boreholes. Parts of each core (0.5 m) were stored at −80 °C (*α*). The bottom part was sectioned through the major axis in two parts, one used for chemical and geochemical characterization (*β*) and the other used for microbiological analysis (*γ*). The image was obtained from Google Map and was modified.

The Panasqueira Mine, located close to the town of Fundão, central Portugal, is owned by Beralt Tin and Wolfram (Portugal) SA (“Beralt”). The actual mining area is known as the “Couto Mineiro da Panasqueira”. This region is characterized by an annual average temperature of 15.1 °C and an average annual rainfall of 1183 mm. Around the mine, there is a pine forest not affected by anthropogenic activity. The slimes resulting from water treatment, as well as the finer fraction of the processing plant are stored together in tailings basins. The mineral processing at the mine changed through the years. The oldest mine tailings basin, Basin 1 (Fig. 1a) was used to store the mine tailings from the opening of the mine until the basin was full and thus closed, in 1985. This basin has approximately, a 750 m perimeter, 35.000 m^2^ in area, 50 m maximum depth, and an elevation of around 600 m. The maximum volume, which is completed, is about 731,600 m^3^, equivalent to 1,817 kt of material^14^. The sediments deposited in this basin were generated during the ore processing but did not included part of the finer fraction. Basin 2 (Fig. 1a) is where the material is now being deposited and has been active since 1985. It has, approximately, a 1 km perimeter, 69,000 m^2^ area, 50 m maximum depth, and an elevation of around 700 m. The maximum volume capacity, which is almost reached, is about 1,545.000 m^3^ equivalent to 3,347 kt of material^14^. Both tailings basins were used in this work for geochemical and biological analysis. A previous geological study^15^ showed that samples collected throughout the Basin 2 were similar in grain size and mineralogical composition. Moreover, differences between basins were also observed with a significant increase in the quartz content from the old fine tailings dam (Basin 1) to the new one (Basin 2).

### Sample collection

Sediment samples were taken from the tailings drill cores collected in Panasqueira mine. All samples were collected on the same day to reduce any heterogeneity imparted by climatic conditions. The sampling was performed in the two basins and four boreholes were drilled (boreholes S4 and S5, in Basin 1, and boreholes S2 and S3, in Basin 2; (Fig. 1a)). Sampling was performed without disturbing the natural conditions of the sediments, such as its structure, texture, density, natural water content, chemistry or stress condition. Undisturbed sediment samples were used to appraise the distribution of microorganisms in different geological conditions, such as physical layers or clusters of minerals. The cores were obtained using a DPT rig able to recover continuous tailings cores through holes barely larger than the core itself, with the absence of cuttings, with a brand new Perspex tube (liner). Right after collection and labeling, the samples were kept in a cooler box at 8 °C, in accordance with the standard guide for direct push soil sampling for environmental site characterizations^16^, and transported to the test laboratory.

Each core was separated into 1 meter (m) sections. The first meter was discarded because the surface would not present stable sediment, and would rather represent a transient community influenced by different discharges and surface aeration and also the texture and dryness of the first meter of sediment did not guarantee a good sediment continuity. The meters 2, 3 and 4 were divided into two halves. The upper 0.5 m (α) of each section was frozen and stored at −80 °C. The bottom 0.5 m was further sectioned into two parts through the major axis. One part (β) was used for chemical and geochemical characterization, while the other (γ) was used for microbiological characterization (Fig. 1b).

### Geochemical analysis

Physicochemical parameters were determined for boreholes S5 and borehole S2 as representatives of Basin 1 and Basin 2, respectively. Each sample was grinded, sieved, powdered to a grain less than 200-mesh and pressed pellets were made for the chemical data obtained by X-Ray Fluorescence with a bench top Analytical Axios mAX spectrometer using a Rh anode X-ray source, at 20 to 60 kV and up to 160 mA. Major elements were read in the Omnian operation mode, and trace elements were read in the Pro-trace operation mode (Table 1). The quality of the data was assessed using duplicate sample analyses and measurement accuracy was estimated at ±5% for all the elements analyzed. pH was determined according to the test method 9045D for soil and Waste pH^17^. Particle size distribution of each original sample was performed by Laser Diffraction method (ISO 13320:2009), using a Master size 2000 equipment from Malvern Instruments Ltd. From each histogram, it was selected D90 [µm] (dimension for which 90% of the particles have an equivalent diameter below this value) as variable that characterizes the particle size. Net Acid Generation (NAG) was quantified in pulverized sample oxygenated by hydrogen peroxide (15%), overnight, and heated for 2 hours or until reaction was complete to remove the remaining H_2_O_2_ and to release the neutralizing particles^18^. The pH of the solution was measured (NAG pH), titrated with NaOH until pH 4.5 then to pH 7. The NAG was calculated in kg of H_2_SO_4_/ton of sample through the mathematical expression: NAG = 49 ∗ V ∗ M/W, where V is the volume of NaOH solution used (ml), M is the molarity of the NaOH solution (mol/L) and W is the weight of the sample used in the test (g). The Total Organic Carbon (TOC) content in the tailings samples was determinate by TOC-VCSN (Shimadzu) coupled with a Solid Sample Module SSM-5000 A (Shimadzu). Tailings samples were also submitted to respirometry tests (ISO 16072:2002) in an OxiTop® Control OC 110 apparatus. Soil respiration (O_2_ consumption and Biochemical Oxygen Demand BOD) were inferred from the pressure measurement in a static system. The system was settled at constant temperature of 25 °C for 15 days.

**Table 1:** Physicochemical parameters measured in sediments collected in the tailings basins.

Chemical Elements (ppm)	Basin 1 - Borehole S5			Basin 2 - Borehole S2		
	2.2	3.2	4.2	2.2	3.2	2.2
F	7410.00	8040.00	8610.00	8080.00	7390.00	7910.00
Na	6209.37	6038.74	6216.79	4970.46	4718.23	5096.58
Al	99588.97	108168.12	107649.45	81848.51	84960.50	84383.62
Si	281515.74	269708.21	270666.46	275186.60	260798.80	271213.36
P	1457.64	1287.44	1370.36	1706.40	1697.67	1418.37
S	7260.34	6111.02	6899.93	17267.84	20167.17	16987.51
K	37439.77	44927.72	41814.66	29902.00	31786.44	30524.62
Ca	3366.24	3616.38	3487.74	4695.58	5660.42	4581.23
Ti	8050.00	8630.00	8440.00	6670.00	6940.00	6760.00
Fe	54282.76	56052.32	54506.58	66243.02	73510.09	68663.04
Sc	14.20	15.70	15.20	11.10	12.10	12.50
V	141.10	161.80	157.00	104.60	99.90	107.40
Cr	110.10	117.50	111.20	88.50	87.80	93.30
Mn	704.40	929.80	868.40	767.00	923.00	801.80
Co	17.20	18.70	17.70	20.40	22.10	23.10
Ni	63.70	62.40	59.20	60.50	67.10	72.00
Cu	2434.80	1475.20	1971.90	2569.90	3582.10	2524.40
Zn	4279.80	3908.40	5106.20	8771.10	11091.30	5960.40
Ga	28.50	29.60	29.70	22.00	23.20	21.40
Ge	15.10	13.70	14.90	7.40	9.40	6.90
As	3667.50	2411.90	3158.00	20126.90	24136.10	22929.90
Rb	531.60	611.90	591.70	397.80	391.20	399.50
Sr	74.40	70.10	81.40	74.80	76.70	67.90
Y	30.80	25.00	26.00	19.90	18.80	19.80
Zr	193.80	195.90	201.60	180.80	165.20	181.90
Nb	12.80	14.30	14.40	9.50	9.60	11.40
Mo	2.80	3.00	2.50	2.30	2.90	6.70
Ag	13.23	14.75	17.35	19.01	19.16	12.01
Cd	58.90	36.60	56.80	109.30	134.60	66.30
Sn	317.50	268.40	344.60	425.70	509.90	289.80
Sb	1.90	4.50	3.50	8.00	9.20	11.10
Cs	76.70	91.30	83.90	40.40	41.70	31.10
Ba	470.20	557.00	544.00	359.80	351.90	374.10
La	29.00	31.90	32.00	20.50	20.00	22.60
Ce	122.10	103.50	107.00	303.70	303.70	315.50
W	1563.30	1370.20	1449.80	942.10	1195.60	1329.60
Pb	130.70	105.50	89.30	90.30	121.30	130.40
**Other variables**						
D90 (µm)	223.12	42.75	66.92	931.62	751.37	554.63
BOD (g/L)	0.15	0.04	0.54	0.19	2.94	0.01
TOC (%)	0.65	0.66	0.71	0.89	0.81	1.05
pH	5.91	5.97	6.05	7.01	6.90	6.54
NAG (kg H_2_SO_4_/t)	7.84	7.64	8.04	16.86	17.44	17.05

D90, Maximum diameter for 90% of the particles; BOD, Biological Oxygen Demand; TOC, Total Organic Carbon in sediments; NAG, Net Acid Generation.

### 16S rRNA gene-based microbiome analysis

#### DNA extraction

The total DNA was extracted by Mannitol-Phosphate Buffer Saline-Cetrimide (Mannitol-PBS-CTAB) method, adapted from Fatima and co-workers^19^, briefly: 1 g of soil sample was weighted and frozen (−80 °C) for 30 min. This was followed by addition of 10 mL of PBS (pH 7.4), vortex to homogenize and shaking for 10 min at 150 rpm (22 °C). The soil suspension was centrifuged at 2200 × *g* for 10 min at 4 °C. The soil sample was washed again with PBS, centrifuged in the same conditions, and resuspended in 10 mL of DNA extraction buffer (200 mM Tris-HCl, pH 8.0; 1 M NaCl; 0.1 M EDTA, pH 8; 0;2% CTAB; 2% SDS; 0.2 M mannitol). The suspension was incubated for 1 hour at 65 °C with occasional stirring. After centrifuging the soil suspension, as previously described, the supernatant was transferred to a new tube and 50 µL of NaCl (5 M) and 50 µL of CTAB (10%) were added.

The tubes were gently shaken by inverting and incubated for 10 min at 4 °C. This was followed by addition of equal volume of chloroform/isoamyl alcohol (24:1) and centrifugation for 30 min at 2200 × *g* at 4 °C. The upper phase was transferred to a new tube and 1/10^th^ volume of sodium acetate (3 M, pH 5.2) and 2 volumes of ethanol 100% were added. The samples were left overnight at 4 °C. After overnight incubation, the suspension was centrifuged as previously described. The supernatant was carefully discarded, the pellet was desalted with ethanol 70% and 100 µL of TE buffer (10 mM Tris-HCl; 1 mM EDTA, pH 8.0) was added. The humic acids were removed from the DNA by adding 200 µL of HTR reagent from E.Z.N.A.® Soil DNA Kit Protocol. The DNA was stored at −20 °C.

#### 16S Amplicon sequencing and bioinformatics pipeline

The 16S rRNA gene amplification was performed using a two-step procedure to amplify the hypervariable V3-V4 region of the 16S rRNA gene, using PCRBIO HiFi Polymerase (PB10.41, PCR BIOSYSTEMS, UK) in 25 µL reactions with 2 µL template. Amplification was performed in 96-well microtiter plates, reactions were run in a 2720 thermal cycler (Applied Biosystems^®^, Life Technologies, CA, US) according to the following cycling program: 1 min of denaturation at 95 °C, followed by 30 cycles of 15 s at 95 °C (denaturing), 15 s at 56 °C (annealing) and 30 s at 72 °C (elongation), final extension at 72 °C for 5 min, and storage at 10 °C thereafter. The PCR products from both steps were purified using High Prep™ PCR (AC-60500, MagBio Genomics Inc., USA) PCR Clean Up System, using 0.65:1 beads to amplicon ratio (vol/vol). The first step used 30 amplification cycles and the modified broad range primers Uni341F (5′-CCTAYGGGRBGCASCAG-3′) and Uni806R (5′-GGACTACHVGGGTWTCTAAT-3′)^20,21^. The second step used 15 amplification cycles and primers developed in-house, which contains sequencing adaptors and unique combinations of forward and reverse indices^22^. A negative template-free control and a positive control containing 2 µl DNA from a known bacterial mock community (1 ng/µl; HM-782D, BEI Resources, VA, US) were included.

Samples were normalized using Sequal Prep Normalization Plate (96) Kit (Invitrogen, Maryland, MD, USA), concentrated using the DNA Clean and Concentrator™-5 kit (Zymo Research, Irvine, CA, USA). The concentration of the pooled libraries was determined using the Quant-iT™ High-Sensitivity DNA Assay Kit (Life Technologies) and adjusted to 1.65 ng/µl (4 nM). Amplicon sequencing was performed on the Illumina MiSeq Desktop Sequencer (Illumina Inc., CA, US), with the denatured libraries adjusted to a final concentration of 16 pM. For each run, a 5% PhiX internal control was included. All reagents used were from the MiSeq Reagent Kits v2 (Illumina Inc., CA, US). Automated cluster generation and 250 bp paired-end sequencing with dual-index reads were performed. The sequencing output was generated as demultiplexed fastQ-files directly on the MiSeq instrument. Up to 192 samples, including controls were sequenced per run.

Sequencing data was analyzed using the Qiime2 (10.7287/peerj.preprints.27295v2) implementation of DADA2 using default parameters^23^ (Supplementary script 1), producing 215,285 high quality merged sequencing reads, representing 1,041 amplicon sequence variants (ASV). The SILVA database version 132 was used for taxonomical classification^24^. The data was imported into R (supplementary script 2: load_data.R) and the taxonomic table was edited to remove uninformative classifications (i.e. metagenome or uncultured) and handle missing information with an R-script (supplementary script 3: clean_taxTable_v2.R). Lastly, ASV classified as Chloroplast, Mitochondria, or without phylum assignation (11 ASV, 55 reads) was removed. Based on the rarefaction curves (Supplementary Fig. S1) it was decided to remove the single sample with less than 2,000 reads.

#### Predictive metagenome analysis

The PICRUSt tool was used to predict the metagenome based on the 16S amplicon data sets^25^. The functional potential of each sample was calculated using the q2-picrust2 implementation tool by G.M. Douglas (https://github.com/gavinmdouglas/q2-picrust2/). The predicted metagenomes were functionally annotated, using the Kyoto Encyclopedia of Genes and Genomes (KEGG) pathways. The functional predictions were assigned to KEGG Orthology (KO) level 3 for all genes. However, the data set was pruned to only include the level 1function: metabolism, environmental information processing, cellular processes and genetic information processing, as the categories of organismal systems and human disease are not relevant in environmental samples. As an indicator for the PICRUSt prediction accuracy, the Nearest Sequenced Taxon Index (NSTI) for each ASV was estimated and calculated per sample.

### Statistical analysis

Statistical analysis and data treatment were performed using the R software platform^26^ (Supplementary script 4: Panasqueira.R.). The R package phyloseq was used for data handling^27^, and all plots were created using the ggplot2 package^28^. The alpha diversity measures, observed richness and Shannon-diversity index (H’), were calculated as the mean of 100 separate rarefactions to 3,397 reads per sample (90% of lowest sample depth). Between groups comparisons of alpha diversity were performed using analysis of variance (function: anova, package: stats). Beta-diversity were calculated as Bray-Curtis dissimilarity index (function: distance, package: phyloseq), using Permutational Multivariate Analysis of Variance using distance matrices (PERMANOVA, function: adonis, package: vegan). To investigate the differential abundance of bacteria, the package DAtest was used to determine which statistical method to employ (function: testDA, package: DAtest) and to perform the suggested tests^29^. Statistical Analysis of Metagenomics Profiles (STAMP) version 2.1.3 software^30^, was used to evaluate the significant differences in the metagenome metabolic profiles between the samples and to functionally visualize the categorized metagenomes generated by PICRUSt. The statistical significance was estimated using G-test (w/Yates’) + Fisher’s, two sided, with p < 0.05 representing statistical significance. The Venn diagram showing the number of taxa shared by, or unique to, the different boreholes sampled was built using the Venn-Diagram free web tool of Bioinformatics & Evolutionary Genomics (http://bioinformatics.psb.ugent.be/webtools/Venn/). Principal Component Analysis (PCA) was determined using CANOCO version 4.5 (Microcomputer Power, Ithaca, NY, USA). This analysis was conducted to assess overall differences between microbial community compositions of the borehole sampled in each basin and correlate them with environmental variables. Data from microbial communities consisted of normalized OTUs (%) and environmental variables consisted of element abundance and physicochemical parameters. Graphical representations along axis PC1 and PC2 had a cumulative percentage variance of 90%. Similarity Percentage Analysis (SIMPER) and Paired *t*-test were applied on the physicochemical parameters using the PAST 3.23 software^31^. The applied SIMPER analysis enabled the identification of physicochemical elements that most contributed to the dissimilarity between the two tailings basins and the Paired *t*-test was used to test whether the differences in physicochemical parameters were significant (P < 0.05).

## Results

### Physicochemical characteristics of the tailings samples

The physical analysis showed that the particles size was higher in Basin 2 with the highest value of 931.62 µm in diameter. Particles with higher diameter were found in upper layers of both basins. The chemical analysis of the two tailings basins of Panasqueira mine showed a very similar composition profile. The most abundant elements in the two basins were Si, Al, Fe, K, S and As (Table 1). The average content of Si was very similar in the two basins while Al and K were significantly higher in abundance in Basin1 (p < 0.05) (Supplementary Table S1). On the other hand, in Basin 2 the content of Fe, S and the hazardous metalloid As were significantly higher (p < 0.05) (Supplementary Table S1). The average content of W was slightly higher in the older tailing Basin 1 (1461 ppm) when compared to the more recent Basin 2 (1156 ppm) (Table 1). Although Cr, Rb and Cs showed differences in concentration between the two basins, they were present in very low abundance. The relative abundance of Ca and Zn were higher, with Ca showing differences statistically significant between the two basins. When comparing the average concentrations of the elements in both basins, the analysis of the variability within each of the basins taking into consideration the estimation of the uncertainties, showed that there is a part of intra basin variability that cannot be fully explained by analytical variability. This is the case of Mn, Cu, Zn, As, Sn and Pb, in Basin 1, and of P, S, Ca and W in Basin 2.

The evaluation of the potential for generation of sulfidic acid (NAG) showed that both tailings basins had acid forming potential, but Basin 1 values, are near the threshold value for low capacity acid production. The total organic carbon (TOC) was low in Basin 2 (0.81–1.05%) and even lower in Basin 1, (0.65–0.71%). The biological oxygen demand (BOD) was positive in all samples and similar in both basins.

### Bacterial diversity of W mine tailings

**Figure 2:**
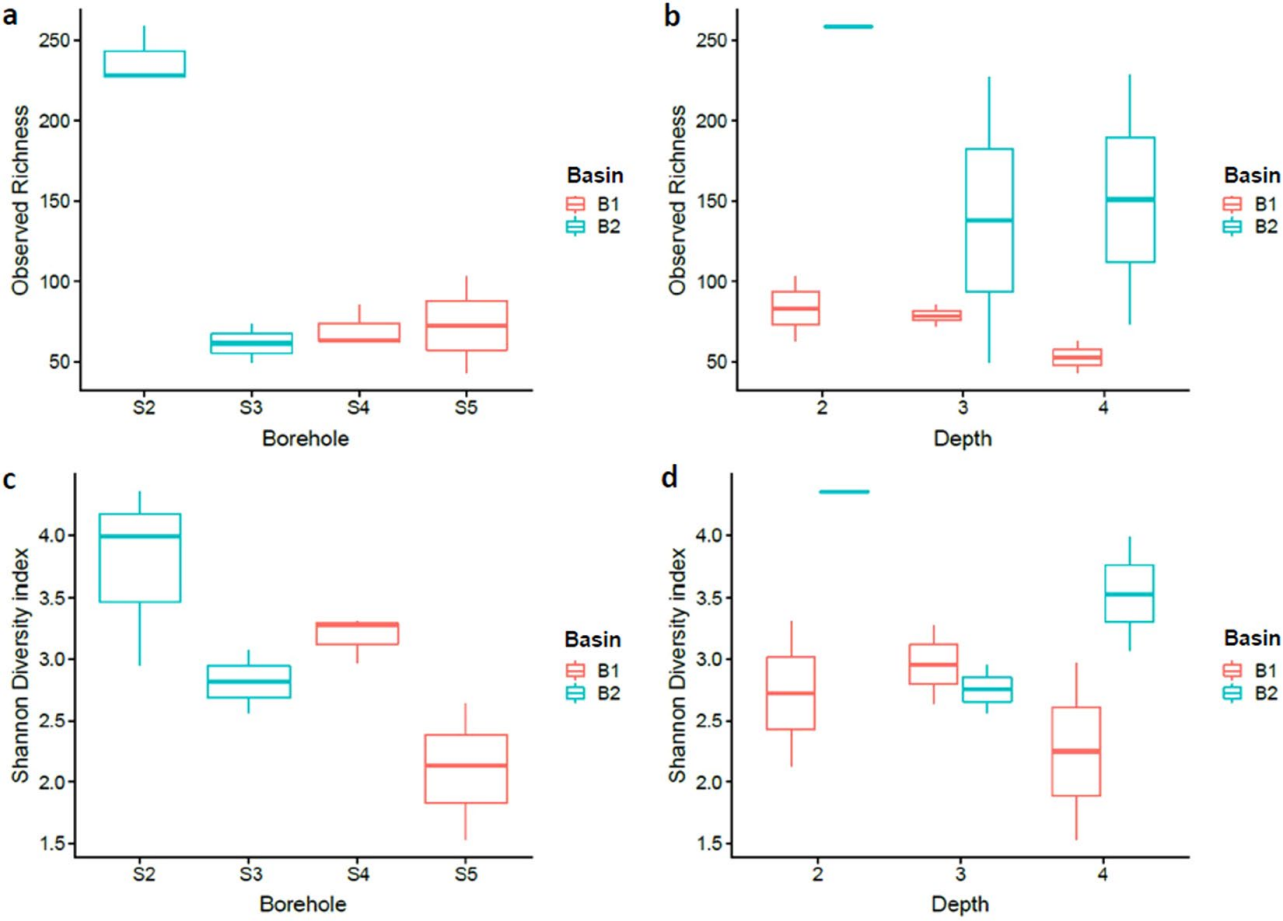
Boxplot of alpha diversity for the sediments of the 4 boreholes determined by observed richness (**a**,**b**) and Shannon Diversity Index (H’) (**c**,**d**). Line in box indicates median value, box covers 95% confidence intervals, vertical lines reaches to furthest samples within 1.5 x box height and any outliers are indicated as points.

A total of 213,230 reads, representing 1,020 unique amplicon sequence variants (ASVs), were generated by sequencing 16S rRNA gene-based amplicons from the 11 samples. The number of reads per sample ranged from 3,775 to 70,194 (mean 19,389 ± 20,125). Borehole S2 has a significantly higher observed richness (mean 238 ± 18) than the other boreholes (mean 61 ± 17–73 ± 30, p < 1.8 * 10^−3^), whereas no differences were seen between borehole S3-S5. While (H’) was highest for borehole S2 (mean 3.8 ± 0.7), only borehole S5 was significantly lower (mean 2.1 ± 0.6, p = 0.023). It was not observed any correlation between the depth at which the samples were taken and alpha diversity (Fig. 2).

### Bacterial composition

**Figure 3:**
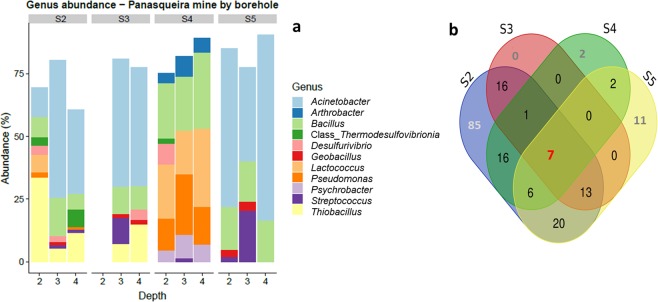
Taxa composition of the four boreholes sampled in Panasqueira mine. (**a**) Bar plot of genera and class abundance within each sample. Samples are sorted by borehole (grey box) and x axis indicate sampling depth. In each sample, genera representing less than 1% have not been plotted. (**b**) Venn diagram showing the number of taxa shared by, or unique to, the different boreholes. Number colored in red represent the number of taxa shared by the four boreholes and numbers colored in grey represent taxa unique to each borehole. Sample S3 at 2 m depth were excluded due to insufficient high quality sequencing reads.

The microbiome in the tailings basins is dominated by *Proteobacteria* (28.8–81.2%, mean: 58.7%) and *Firmicutes* (12.1–69.2%, mean: 33.7%), together represent 80.1–99.2% of the microbiome in each sample, with samples from borehole S4 having the highest proportion of *Firmicutes* (Supplementary Fig. S2). On genus level, there was a larger variation between the boreholes (Fig. 3a). Borehole S2, S3 and S5 were all dominated by *Acinetobacter* (46.6%), followed by *Bacillus* (12.4%). In borehole S5, at 2 and 3 m depth, *Streptococcus* were also found (2.1% and 20.5%, respectively) and to a lesser extent, *Geobacillus* (2.8% and 3.6%, respectively). In borehole S3 *Thiobacillus*, *Streptococcus*, *Geobacillus*, and *Desulfivibrio*, combined, represented 19.2% and 21.6%. In borehole S2, samples from 3 and 4 m were similar to that of borehole S3, while the sample taken at 2 m depth contained a high proportion of *Thiobacillus* (33.6%) as well as *Lactococcus* (7.2%). Borehole S4 had a very different composition, with almost no *Acinetobacter* (0.4%), dominated by *Bacillus* (24.7%), *Lactococcus* (23.4%), and *Pseudomonas* (17.4%), with lower amounts of *Psychrobacter* (6.9%) and *Arthobacter* (5.8%).

One family and six genera composed the core microbiome of the tailings sediments of the Panasqueira mine, defined by the Venn diagram analysis of the four boreholes (Fig. 3b). The strains from the family *Anaerolineacea* are chemoorganoheterotrophic fermenter bacteria isolated mostly in anaerobic environments. The six genera common to all boreholes are comprised of the two most abundant genera *Acinetobacter* and *Bacillus* and by the genera *Pseudomonas*, *Streptococcus*, *Cellulomonas*, and *Rothia*. Members of these genera include heterotrophic, facultative anaerobic genera commonly found in soil microbiome.

**Figure 4:**
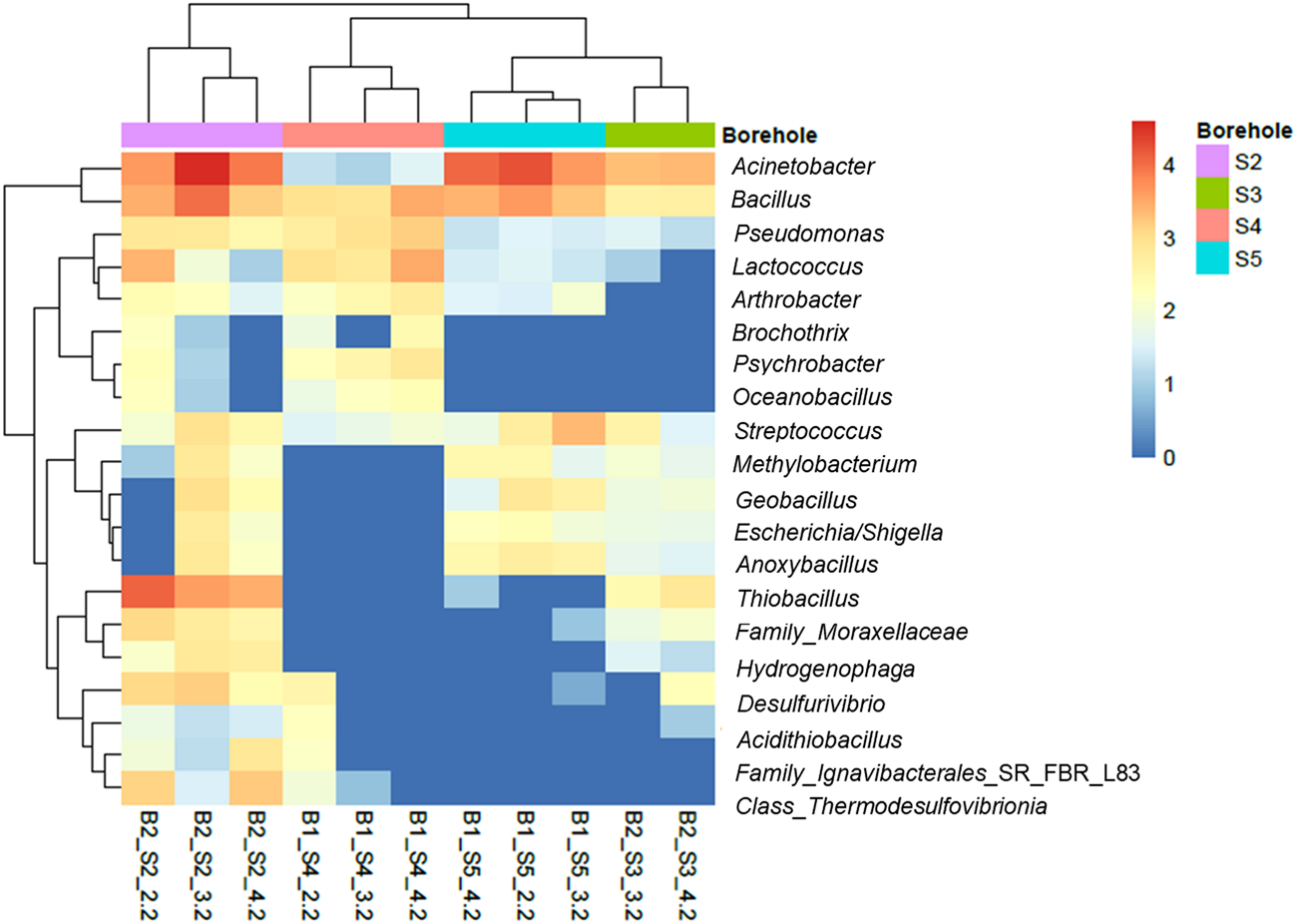
Heatmap of the 20 most abundant genera. Samples are ordered using Ward clustering following Ward’s clustering criterion. Top color bar indicates borehole, the colors in the heatmap indicate the log-transformed (log(0) = 0) read count. Heatmap generated using the R-package pheatmap.

To provide a clearer view of the similarity and grouping between samples a heatmap was designed for the 20 most abundant bacterial genera (Fig. 4). The boreholes S3 and S5 were characterized by the absence of *Lactococcus*, *Arthrobacter*, *Brochothrix*, *Psychrobacter* and *Oceonobacillus* that were part of the microbiome of borehole S4 and the first meters of borehole S2 (Fig. 4). On the opposite, the genera *Methylobacterium*, *Geobacillus*, *Escherichia*-*Shigella* and *Anoxybacillus* were absent in borehole S4 and upper borehole S2 and present in the others. A biomarker of borehole S2 was the presence of *Thiobacillus* that was also found in very low levels in borehole S3. Some of the OTUs were not identified at the genus level and included organisms of the class *Thermodesulfovibrionia*, and family *Ignavibacteriales*_SR_FBR_L83 and *Moraxellaceae*, which were present mainly in Basin 2.

### Linking the microbial communities to the physicochemical parameters of the tailings basins

**Figure 5:**
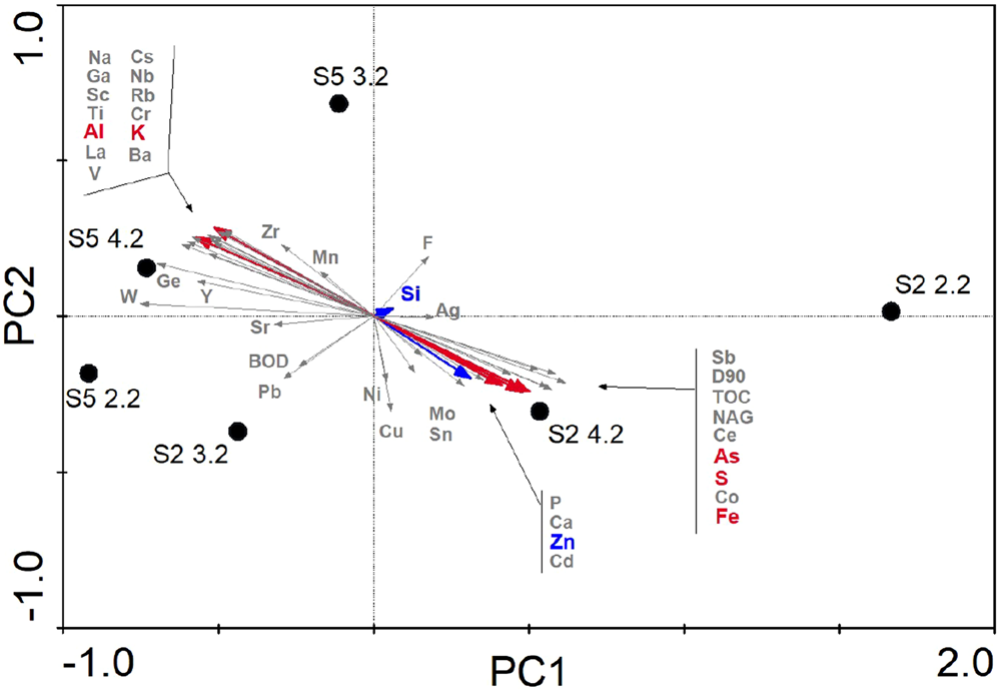
Principal Component Analysis (PCA) of OTU composition of boreholes S5 and S2, and correlation with physicochemical parameters determined in each borehole. Borehole S5 and Borehole S2 is representative of Basin 1 and Basin 2, respectively. Colored elements represent the most abundant ones (>10.000 ppm in at least one sample) of these, elements colored in red represent those that are responsible for more than 10% of the dissimilarities between basins by SIMPER analysis (Supplementary Table S2).

The PCA plot was used to show the correlation of the physicochemical parameters with microbial OTU composition of the boreholes S5 (Basin1) and S2 (Basin 2) (Fig. 5). The PCA results showed that the first (PC1) and the second axis (PC2) explained 77.4% and 12.6% of the total variance, respectively. This analysis showed that the microbial communities of borehole S5 from Basin 1 grouped together and that the microbial communities of borehole S2 from Basin 2 were more distinct, in particular, the upper layer one. Of the most abundant chemical elements (>10.000 ppm), Al-K were highly correlated with the microbial community of borehole S5, while As-S-Fe-Zn were correlated with samples of borehole S2. Except for Zn, all of these elements contributed more than 10% to the observed differences in the chemical composition between the two basins by SIMPER analysis (Supplementary Table S2).

### Functional profiling of the soil microbial community

The PICRUSt metagenome predictions had NSTI scores ranging from 0.00733 to 0.0739, for the 4 boreholes sampled, implying that the predicted metagenomes were reliable for subsequent functional analysis of the communities^25^.

**Figure 6:**
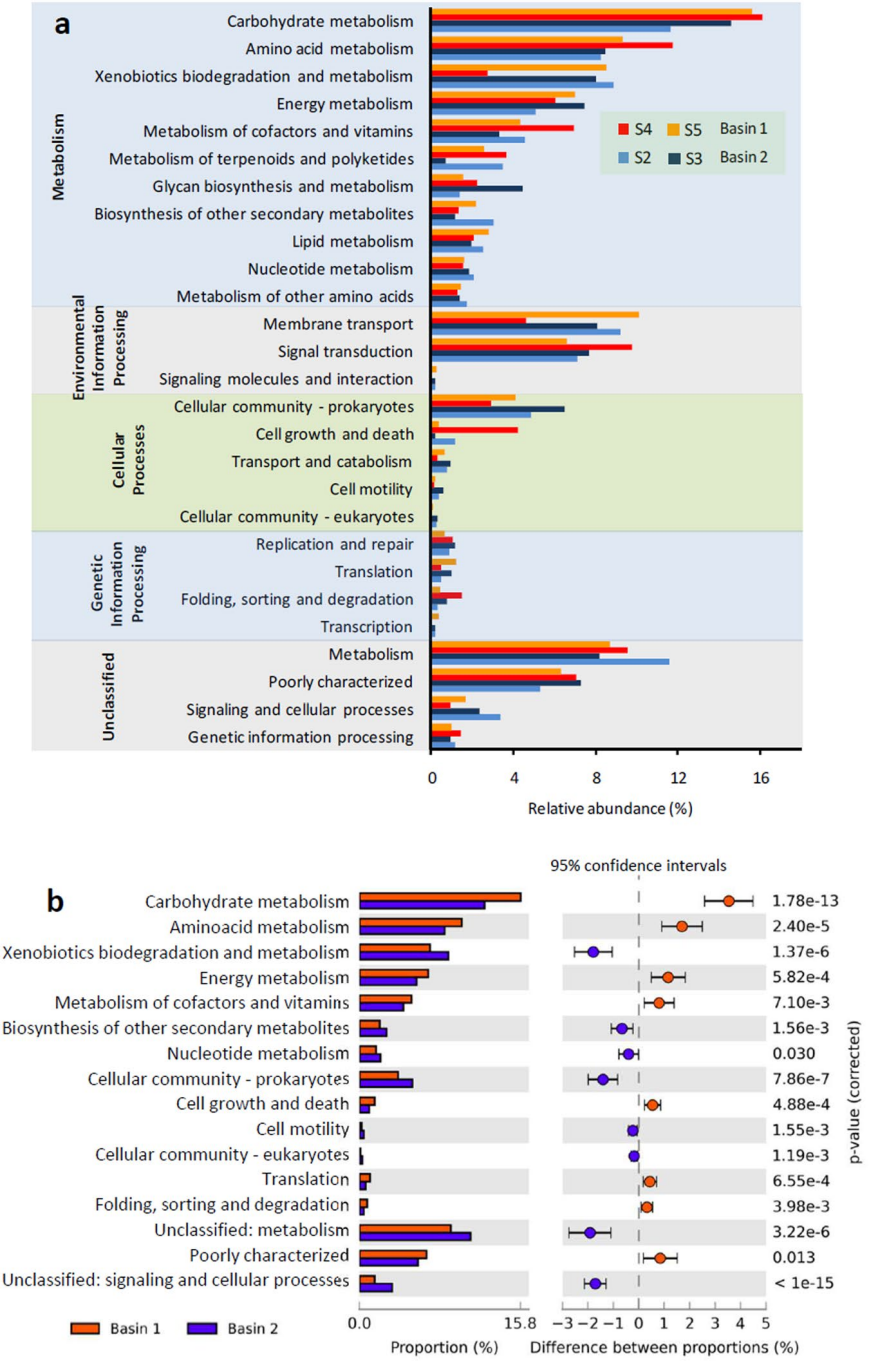
PICRUSt function analysis. (**a**) The relative abundance of the predicted functions grouped according KEGG level 1 and 2 categories of the bacterial communities in the four boreholes sampled in Panasqueira mine. (**b**) Predicted functional subsystems at KEEG level 2 significantly different (P < 0.05) between the Basin 1 and Basin 2 using STAMP software.

The most abundant KEGG pathways predicted by PICRUSt in the 4 boreholes are summarized in (Fig. 6a). These principal metabolic pathways were common to all samples analyzed here. Considering the total genes, 52.7 to 57.2% were related to the metabolism pathways (Fig. 6a). The genes families related to this category showed significant abundance differences (G-test (w/Yates’) + Fisher’s, two-sided, P < 0.0.5) between the 2 basins, specifically those related to the carbohydrate, amino acid and energy metabolism, and also to xenobiotics biodegradation and to the metabolism of cofactors and vitamins (Fig. 6b).

Gene families related to the environmental information processing were also abundant (14.5 to 16.9%) in the two tailings basins. Among these, genes involved in membrane transport and signal transduction were predominant, accounting for 5 to 10% of all genes.

Genes involved in cellular processes (5.5 to 7.4%), especially those related to cellular community-prokaryotes showed significant differences between the two basins (Fig. 6b). High representations of unclassified gene families (17.7 to 21.4%) were also present in the microbial communities of both basins.

The microbiome of Basin 1 had a predicted carbohydrate metabolism more directed to the use of fructose and mannose, and to the use of pyruvate, when compared to the microbiome of Basin 2 (Supplementary Fig. S3, p = 8.55 * 10^−15^ and p = 1.99 * 10^−7^, respectively). The most abundant energy metabolic pathway in the two basins was the methane metabolism, although more abundant in Basin 1. Several amino acid synthesis pathways were predicted, of which the most prominent in the Basin 1 were the tryptophan and tyrosine synthesis while in Basin 2, cysteine and methionine, glycine, serine and threonine synthesis pathways were more abundant (Supplementary Fig. S3). Genes related to degradation of aminobenzoate and benzoate were the more abundant within the pathways of xenobiotics degradation and metabolism. The microbiomes of both basins showed genes for most of KEGG pathways related to the prokaryotic cellular community. Differences between basins were predicted with Basin 2 showing a higher percentage of genes in these pathways (Supplementary Fig. S3).

## Discussion

This study describes the microbiome structure analysis of tailings from a tungsten mine (Panasqueira mine). To date, there are no other reports on this type of tailings. Integrated surveys were employed such as geochemical analyses and microbial diversity by high-throughput sequencing to predict metabolic information about the microbiome in tailings. Previous studies suggested that environmental factors are key elements to shape microbial profiles in mine-related wastes and includes, metal concentration, temperature, pH, dissolved oxygen, and total organic carbon^6,32^. Sediments with low organic carbon content, as found in Panasqueira tailings, are a common characteristic of these environments, also reported by other studies^33–35^. This is not surprising since the tailings basins are lacking either plant nutrients or vegetation growth. Therefore, exposure to high concentrations of metals and low carbon for extended periods selects for microorganisms able to deal with such harsh environments.

The chemical composition of the tailings was very similar in both basins. Nevertheless, higher concentrations of As, Fe and S were found in Basin 2, while K and Al concentrations were higher in Basin 1. Arsenic and Cd, the base metal Zn and the major element S are 7, 2, 2 and 3 fold higher in Basin 2 when compared to Basin 1. The tailings result of ore processing and the two basins reflect two different management of W recovery process, used by the company at different times. The sediments of Basin 1 are the result of a less refined and less efficient mining process, resulting in a higher W concentration in the discarded material. Besides, Basin 1 did not receive the finer sediments rich in arsenic being deposited in another area (Internal report Minas da Panasqueira).

Basin 2, more recent, apart from sand and slimes, receives sludge from the water treatment plant, as well as the copper circuit tailings containing arsenic. These differences shaped the microbial communities of the tailings. Low microbial diversity and species richness were found in both basins. However, there are differences in microbial diversity, which seemed to be related to the tailings basins origins and characteristics. Higher diversity in the newest basin could be related to the fact that this basin is still receiving residues and wastewater, containing more carbon, sulfur, and iron.

Samples collected at the same borehole showed similar microbial composition and diversity at different depths, which was expected considering that a chemical stratification was not visible in both basins.

One of the main features that determine the community composition in an environment is its tolerance to stress factors. Modifications in the tolerance or modifications of selective environmental conditions will cause shifts in community composition, with well-adapted species replacing less adapted ones^36^. Previous works in mine tailings of different chemical compositions (mostly acid-generating mine residues) included mainly *Actinobacteria*, *Proteobacteria* and *Firmicutes*^37,38^. The predominance of these groups varied according to the metal composition of tailings or residual waters^34,39,40^. In Panasqueira mine, all these groups were present, but *Actinobacteria* constituted a minor group. The low abundance of this group, together with the presence *Nitrospirae* and *Bacteroidetes* could be related to the origin of these tailings being a tungsten mine (sediments with less potential for sulfidic acid generation, and pH 6–7)^41^. The bacteria from phylum *Proteobacteria* are ubiquitous in nature mostly due to their capabilities to cope with hostile life conditions such as extreme pH, oligotrophic environments and metal-rich environments^39^. The main representative of this group and part of the core microbiome of both tailings basins was *Acinetobacter*, a genus known to include organisms with high genetic plasticity and diversity of resistance mechanisms^42^. The microbiome of Basin 2 characteristically had a major population *Thiobacillus* that was not present in Basin 1. This may be related to the different amounts of S between basins even though Fe content was similar. The abundance of Fe and S in Basin 2 could be used by these chemolithotrophs as a source of nutrients. Members of the genus *Thiobacillus* can oxidize ferrous and sulfur compounds and play an important role in Fe and S cycling^43,44^. This group of bacteria also has a high tolerance to several metal ions^45^. The *Lactococcus*, *Arthrobacter* and *Psychrobacter* microbial composition of borehole S4 (Basin 1) has also been found in tailings from a copper mine^42^.

Given the very low levels of organic carbon across the tailings sites, a selection for chemolithoautotrophic organisms would be expected, considering the data available from pyrite mines. However, many different carbon metabolisms were predicted. Among them, the ones related to fructose and mannose metabolism and also with the amino sugar and nucleotide metabolism were the most abundant. This suggests that in these microbiomes, carbon metabolism diversity was selected instead of inorganic carbon fixation ability as a strategy to overcome the limitation of carbon. Moreover, the microbiomes of the two basins also included bacterial genera with preferential heterotrophic metabolism. The presence of hydrocarbons at low concentration, mainly in the tailings of Basin 2, originating from ore processing (Internal report Minas da Panasqueira) may also contribute to the selection of metabolisms.

The predicted amino acid metabolism also played an important role in these microbiomes. Higher sulfur content in Basin 2 might explain a higher predictive abundance of genes involved in the synthesis of amino acids cysteine and methionine. On the other hand, the microbial community of Basin 1 had a predicted higher enrichment of genes related to the synthesis of aromatic amino acids like tyrosine and tryptophan. The microbial communities of both basins also exhibited a high abundance of genes related to the xenobiotic biodegradation and metabolism. Some studies suggest an association between xenobiotic degradation genes and xenobiotic biodegradation rates^46,47^, and that these functional genes could be used as indicators of the presence of xenobiotic and their metabolites^48–50^. The presence of xenobiotic compounds in the tailings basins could be related to the mining and ore processing in Panasqueira mine. The most abundant energy metabolic pathway predicted for the bacterial communities was the methane metabolism, although more abundant in Basin 1. Based on the predicted functions of the bacterial communities methane oxidation is expected since the *pmo-amo* and *mdh* set of genes were predicted and contribute to methane catabolism^51^. On the other hand, the production of methane was also expected since a set of genes *(mvh*, *mtr* and *mtd*) involved in different steps of this process were predicted in these microbiomes^52^. Members of the family *Anaerolineacea* that are part of the core microbiome are known syntrophs of methanogenic anaerobic bacteria using several carbon sources and proteins^53^. Genes related to nitrogen metabolism were not predicted as abundant in these microbiomes. Several studies have demonstrated that nitrogen fixation is highly affected in metal-contaminated habitats, which is also visible on the ability of nitrogen-fixing bacteria to survive in these conditions^54–56^.

The Panasqueira microbial community was rich in genes related to environmental information processing as signal transduction and membrane transport. Membrane transporters are usually involved in mechanisms of metal resistance in bacteria^57,58^ or antibiotic resistance that can be related to the community mobilome^59^. Levels of arsenic, an acute toxic metalloid, are considerably high in Panasqueira mine, in particular at Basin 2. The dominant environmental forms of arsenic are arsenate and arsenite and these elements are of natural occurrence in waters, soils, and minerals^60^. However, many microorganisms can thrive in such sites, by developing mechanisms for arsenic resistance. The predicted functions of the microbial communities included genes related to arsenic resistance. These genes were associated with arsenite oxidation by the presence of *aoxB* genes^61^, and arsenate reduction and arsenite extrusion by *arsBC* set of genes^57^.

Microorganisms that grow in metal-rich tailings are important players in the process of bioleaching of low-grade ores and show potential for metal mobilization and immobilization^8^. Strategies such as selective metal accumulation through membrane transporters, the production of metal chelating biopolymers and the production of siderophores for selective metal capture were identified in strains of the genera that compose the microbiome^13,36^. These microbial strategies can be used in bioremediation of heavy metal polluted sites but also in metal recovery^62^. Understanding these strategies will allow exploring tailings and mine residues as secondary sources of raw materials. This can be achieved through *in situ* bioaugmentation treatment of the tailings, using selected autochthonous groups of microorganisms with the ability to interact with metals and elements of interest^61^. An in-depth understanding of the tailings microbiome and its putative metabolic capabilities can provide, therefore, a direction for the management of tailings disposal sites and processes.

This study concludes that the metal composition of the basins containing mine tailings reflect the different managements used in ore processing. These mine tailings harbor a diverse and unique microbial community and the distribution of the different microbial groups is related to the physicochemical characteristics of the tailings. The predicted functional profiles of the bacterial communities of the two tailings basins are similar, notwithstanding their taxonomic heterogeneous compositions. This observation should take into account that the accuracy of PICRUSt analysis on predicted microbiome functions depends on the level of correspondence between 16S rRNA gene identification and the sequenced genomes available in the databases. Finally, the microbial community structure present in these mine tailings can be an important reservoir of microorganisms with biotechnological potential.

## Supplementary information

Dataset 1

## Data Availability

The obtained sequence data of this study were deposited in Sequence Read Archive (SRA) under BioProjectIDPRJNA527255. All other data generated or analyzed during this study are included in this published article and its supplementary information files.
