# Supplementary material for: Tailings microbial community profile and prediction of its functionality in basins of tungsten mine
Source: Sci Rep. 2019 Dec 20;9:19596. doi: 10.1038/s41598-019-55706-6 (PMC6925229; doi:10.1038/s41598-019-55706-6)
Supplement: Supplementary file 1 — Dataset 1 [file 41598_2019_55706_MOESM1_ESM.pdf]

Tailings microbial community profile and prediction of its functionality in basins of tungsten mine

Ana Paula Chung[1], Carina Coimbra[1], Pedro Farias[1], Romeu Francisco[1], Rita Branco[1], Francisco V. Simão[2#], Elsa Gomes[2], Alcides Pereira[2], Maria C. Vila[3], António Fiúza[3], Martin S. Mortensen[4], Søren J. Sørensen[4] and Paula V. Morais[1]*

[1]Centre for Mechanical Engineering, Materials and Processes and Department of Life Sciences, University of Coimbra, 3000-456 Coimbra, Portugal.

[2]Centre for Earth and Space Research and Department of Earth Sciences, University of Coimbra - Pólo II, 3030-790 Coimbra, Portugal.

[3]Department of Mining Engineering and Research Centre on Environment and Natural Resources, Faculty of Engineering, University of Porto, 4200-465 Porto, Portugal

[4]Section of Microbiology, Department of Biology, University of Copenhagen, 2100 Copenhagen, Denmark

* Corresponding author: Paula V. Morais,

e-mail address: pvmorais@ci.uc.pt

Francisco V. Simão present address: Research Centre for Economics and Corporate Sustainability (CEDON), Department of ECON-CEDON, Katholieke Universiteit Leuven (KU Leuven), Brussels Campus.

Supplementary Materials

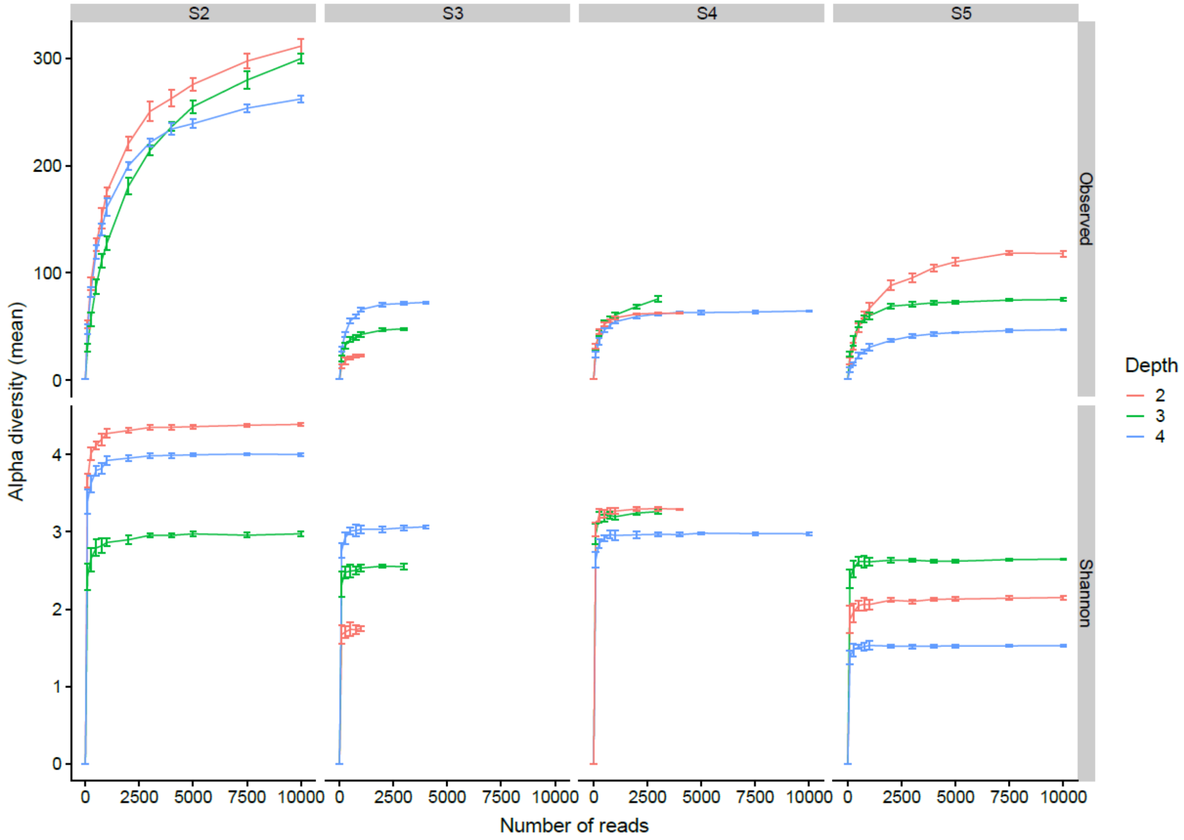

Supplementary Figure S1. Rarefaction curves separated by borehole and colored by depth. Top row is observed richness while the lower is Shannon diversity index. Error bars indicate SD of 10 separate rarefactions for each number of reads within the individual samples.

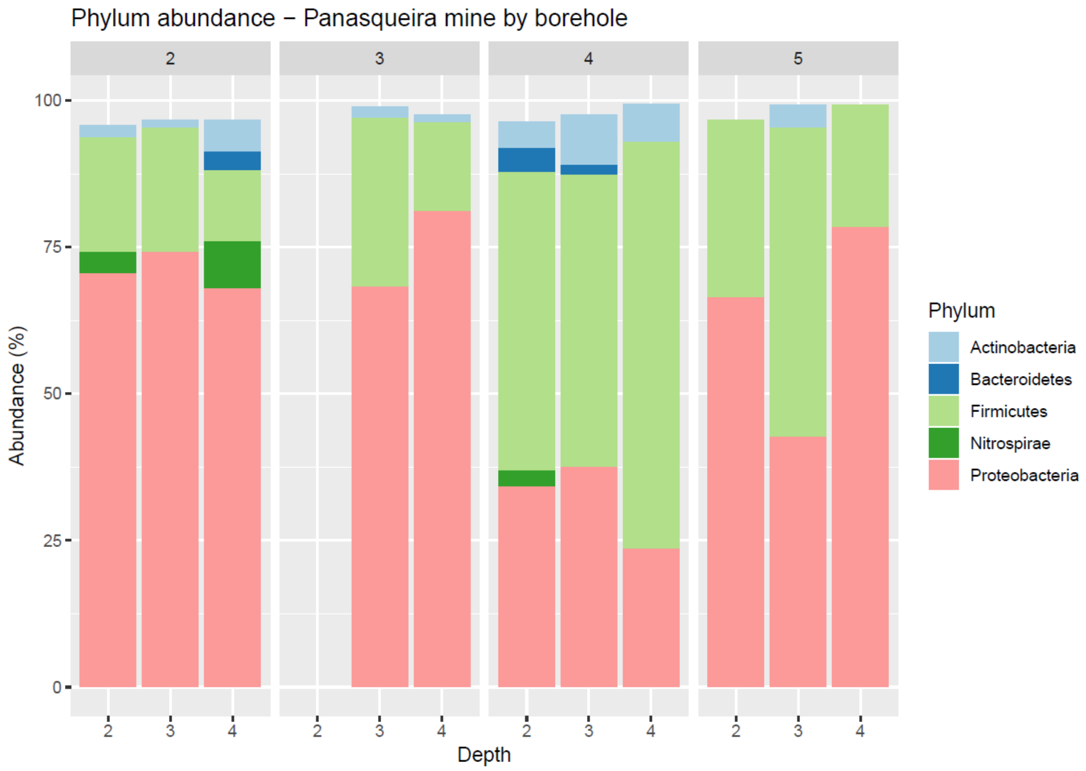

Supplementary Figure S2. Barplot of the phylum abundance within each sample. Samples are sorted by borehole (grey box) and x axis indicate sampling depth. In each sample, phyla representing less than 1% have not been plotted.

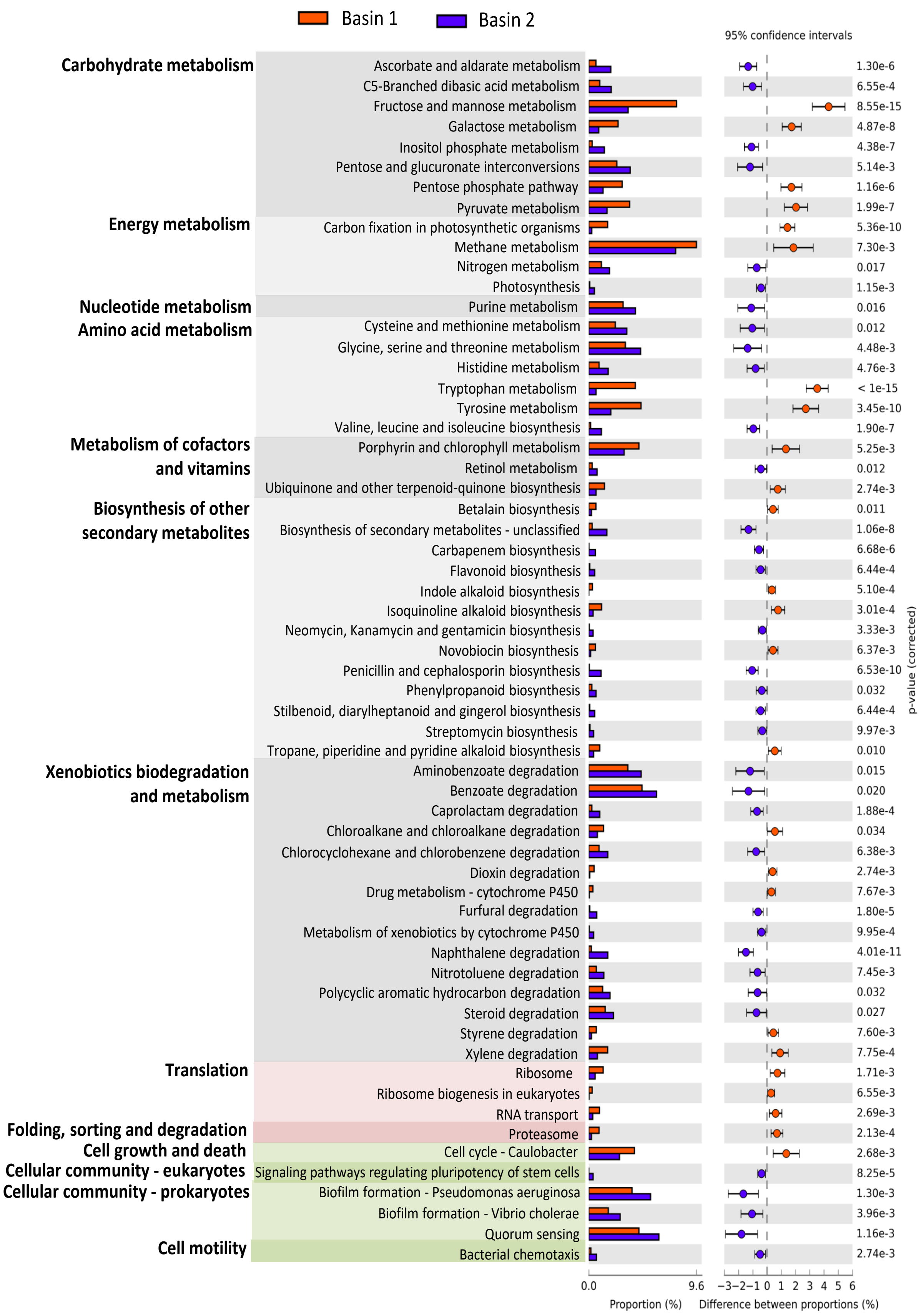

Supplementary Figure S3. Relative abundances of predicted functions (KEGG level 2 and 3) of the microbial communities in the two basins of Panasqueira mine using PICRUSt. Only predicted function that are significantly different in abundance between the two basins are represented ($P < 0.05$).

Supplementary Table 1. Paired *t*-test of physicochemical parameters measured in sediments collected in the tailing basins. D90, Maximum diameter for 90% of the particles; BOD, Biological Oxygen Demand; TOC, Total Organic Carbon in sediments; NAG, Net Acid Generation

Chemical Elements	Basin 1 - Borehole S5			Basin 2 - Borehole S2			Paired *t*-test
	Mean abundance (ppm)	SD	N	Mean abundance (ppm)	SD	N	*p* value
F	8020.00	600.25	3	7793.33	359.49	3	0.664
Na	6154.97	100.72	3	4928.42	192.65	3	**0.002**
Al	105135.50	4810.45	3	83730.88	1655.50	3	**0.007**
Si	273963.50	6558.02	3	269066.30	7430.33	3	0.225
P	1371.81	85.11	3	1607.48	163.83	3	0.153
S	6757.10	587.82	3	18140.84	1760.44	3	**0.014**
K	41394.05	3761.65	3	30737.69	960.12	3	**0.023**
Ca	3490.12	125.09	3	4979.08	592.82	3	**0.035**
Ti	8373.33	295.69	3	6790.00	137.48	3	**0.004**
Fe	54947.22	963.57	3	69472.05	3700.47	3	**0.012**
Sc	15.03	0.76	3	11.90	0.72	3	**0.007**
V	153.30	10.83	3	103.97	3.79	3	**0.022**
Cr	112.93	3.99	3	89.87	2.99	3	**0.023**
Mn	834.20	116.53	3	830.60	81.89	3	0.932
Co	17.87	0.76	3	21.87	1.37	3	**0.030**
Ni	61.77	2.32	3	66.53	5.77	3	0.411
Cu	1960.63	479.90	3	2892.13	597.96	3	0.261
Zn	4431.47	613.13	3	8607.60	2569.35	3	0.151
Ga	29.27	0.67	3	22.20	0.92	3	**0.008**
Ge	14.57	0.76	3	7.90	1.32	3	**0.030**
As	3079.13	631.50	3	22397.63	2056.92	3	**0.006**
Rb	578.40	41.77	3	396.17	4.38	3	**0.019**
Sr	75.30	5.70	3	73.13	4.63	3	0.751
Y	27.27	3.10	3	19.50	0.61	3	**0.038**
Zr	197.10	4.04	3	175.97	9.34	3	0.055
Nb	13.83	0.90	3	10.17	1.07	3	**0.019**
Mo	2.77	0.25	3	3.97	2.39	3	0.509
Ag	15.11	2.08	3	16.73	4.09	3	0.689
Cd	50.77	12.31	3	103.40	34.53	3	0.175
Sn	310.17	38.63	3	408.47	111.06	3	0.370
Sb	3.30	1.31	3	9.43	1.56	3	**0.018**
Cs	83.97	7.30	3	37.73	5.78	3	**0.012**
Ba	523.73	46.81	3	361.93	11.25	3	**0.028**
La	30.97	1.70	3	21.03	1.38	3	**0.010**
Ce	110.87	9.88	3	307.63	6.81	3	**0.001**
W	1461.10	97.04	3	1155.77	196.80	3	0.194
Pb	108.50	20.86	3	114.00	21.02	3	0.841
**Other variables**							
D90 (µm)	110.93	97.91	3	745.87	188.56	3	**0.013**
BOD (g/L)	0.24	0.26	3	1.04	1.65	3	0.530
TOC (%)	0.67	0.03	3	0.92	0.12	3	**0.047**
pH	5.98	0.07	3	6.82	0.25	3	**0.044**
NAG (kg H_2SO_4/t ore)	7.84	0.20	3	17.12	0.30	3	**0.001**

Supplementary Table 2. Similarity Percentage analysis (SIMPER) of the contribution (~75% of total) of the different chemical elements to the average dissimilarity between the two tailing basins in terms of physicochemical characteristics

Chemical elements	Average Dissimilarity	Contribution (%)	Cumulative (%)	Mean Abundance (%)	
				Basin 1	Basin 2
Al	2.02	22.08	22.08	20.04	15.61
As	1.82	19.92	42.00	0.59	4.18
Fe	1.37	14.97	56.97	10.48	12.96
S	1.07	11.74	68.70	1.29	3.38
K	1.01	10.99	79.69	7.90	5.73
